# Safety in glomerular numbers

**DOI:** 10.1007/s00467-012-2169-x

**Published:** 2012-04-25

**Authors:** Michiel F. Schreuder

**Affiliations:** Department of Pediatric Nephrology, 804, Radboud University Nijmegen Medical Centre, PO Box 9101, 6500 HB Nijmegen, The Netherlands

**Keywords:** Kidney development, Glomerular hyperfiltration, Nephron endowment, Solitary functioning kidney, Intrauterine growth restriction, Prematurity

## Abstract

A low nephron number is, according to Brenner’s hyperfiltration hypothesis, associated with hypertension, glomerular damage and proteinuria, and starts a vicious cycle that ends in renal failure over the long term. Nephron endowment is set during foetal life, and there is no formation of nephrons after 34–36 weeks of gestation, indicating that many factors before that time-point may have an impact on kidney development and reduce nephron numbers. Such factors include maternal malnutrition, stress, diseases, such as diabetes, uteroplacental insufficiency, maternal and neonatal drugs and premature birth. However, other congenital anomalies, such as renal hypoplasia, unilateral renal agenesis or multicystic dysplastic kidney, may also lead to a reduced nephron endowment, with an increased risk for hypertension, renal dysfunction and the need for renal replacement therapy. This review focusses on the causes and consequences of a low nephron endowment and will illustrate why there is safety in glomerular numbers.

•

## Introduction

**Fig. 1:**
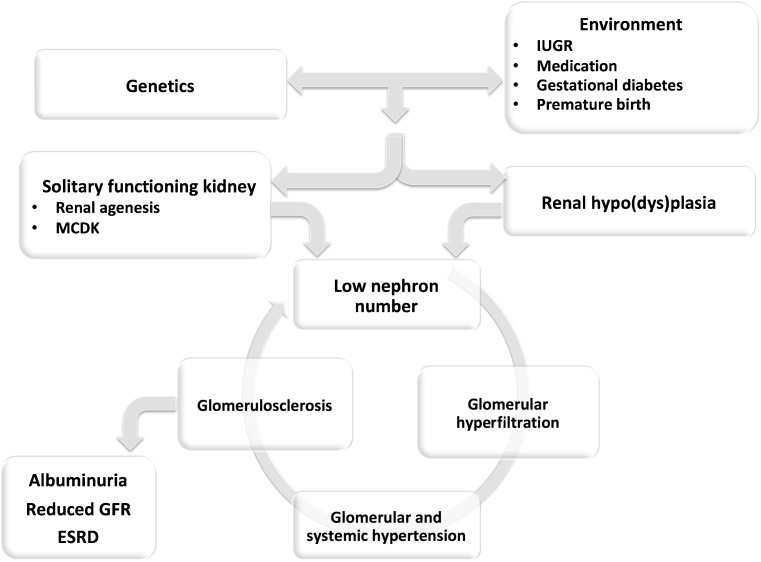
Integration of causes and consequences of low nephron endowment due to programming of kidney development (**upper**) leading to glomerular hyperfiltration (**lower**).* MCDK* Multicystic dysplastic kidney,* IUGR* intrauterine growth restriction,* GFR* glomerular filtration rate,* ESRD* end-stage renal disease

With the hyperfiltration hypothesis, Brenner et al. showed that renal mass reduction results in glomerular alterations that may have adverse effects in the long run [1]. The compensatory glomerular hyperfiltration is associated with glomerular hypertension and enlargement at first, followed by systemic hypertension, proteinuria and glomerulosclerosis. This starts a vicious cycle, with a further reduction in renal mass that results in an on-going decline in glomerular filtration rate (GFR) and may end in chronic kidney disease. The hyperfiltration hypothesis therefore indicates that there is safety in glomerular numbers. As this number is set during nephrogenesis, unrestricted kidney development resulting in nephron endowment is vital for future health. This review considers some of the environmental influences that have been shown to be important in determining nephron numbers and the long-term effects of reduced renal development (Fig. 1).

## Kidney development

Nephrons are formed through the mutual induction of the ureteric bud and the metanephric mesenchyme. This process starts around the fourth to fifth week of gestation, and, more importantly, finishes around the 34–36th week of gestation [2]. When complete, there is no possibility of forming new nephrons later in life, indicating that the nephron endowment an individual is born with will have to last his/her entire lifetime. After completion of nephrogenesis, an average of little under 1 million nephrons per kidney have been formed [3].

There are several ways in which kidney development goes off track, all of which lead to congenital anomalies of the kidney and urinary tract (CAKUT), ranging from renal agenesis to hypoplasia to dysplasia [4]. Genetic but also environmental factors have been associated with the development CAKUT. For instance, a multicystic dysplastic kidney (MCDK) is more frequently seen in offspring from women with diabetes or following the use of certain anti-epileptic drugs during pregnancy, but is also associated with genetic mutations in genes such as* HNF1beta* [5]. CAKUT is frequently identified during prenatal ultrasound screening. However, subtle alterations, such as a low nephron endowment, may not be picked up as it is not yet possible to count nephrons in vivo. This may well be achieved in the coming decade, as magnetic resonance imaging of individual glomeruli in vivo is within reach [6].

## Nephron numbers

A so-called hypoplastic kidney, indicating a small size on ultrasound, is supposed to have a reduced number of nephrons. Even though studies have presented an association between nephron numbers and renal weight [7], only a small part (about 10 %) of the variation in nephron numbers is explained by a difference in renal size in adulthood (M.F. Schreuder, unpublished data). In addition, there is an almost tenfold variation in normal nephron numbers, ranging from a little over 200,000 to over 2.5 million nephrons per kidney [3]. Some of this variation may be explained by genetic differences, and many environmental factors may influence final nephron numbers as well [8].

The relevance of the variation in nephron endowment is evident from the hyperfiltration hypothesis [1]. And indeed, a low nephron number has been found in patients with hypertension referred to as “essential” hypertension. Keller and colleagues showed in a group of victims of motor vehicle accidents that the ones with hypertension had only about half the number of nephrons than their respective matched controls with normal blood pressure [9]. Interestingly, there was no difference in the weight of the kidneys between the groups. In another study, Hughson et al. showed that there is an inverse relationship between nephron number and blood pressure, but only in Caucasians [10]. No such association was found in African Americans, but a higher blood pressure persisted with a higher nephron number. The question remains as to why a normal nephron endowment in African Americans does not result in a normal blood pressure and illustrates that additional factors to nephron numbers determine blood pressure [11].

## Solitary functioning kidney

**Fig. 2:**
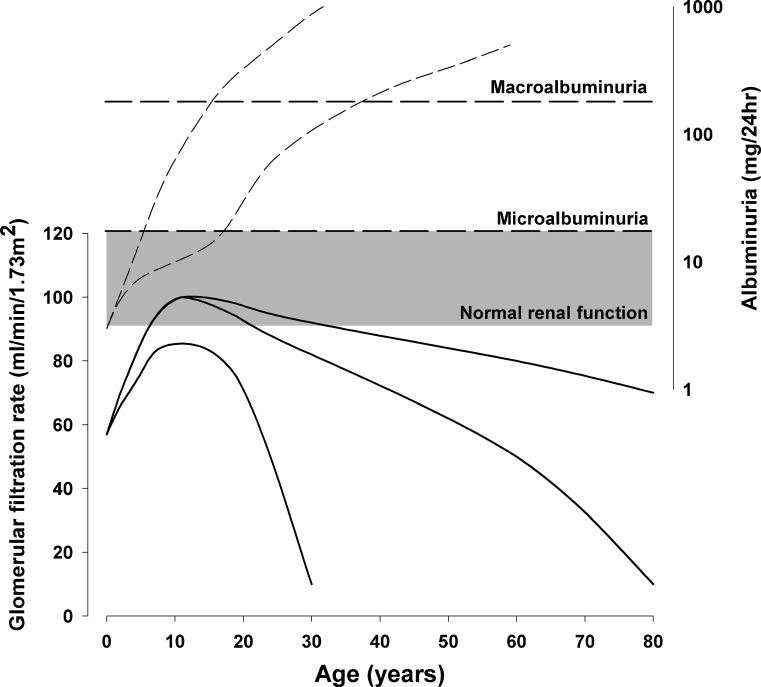
Different potential courses of albuminuria and glomerular filtration rare (GFR) in individuals with a congenital solitary functioning kidney, based on an integration of data from previous studies [13, 14, 16]. GFR (*solid lines*) may mature to normal two-kidney values (90–120 ml/min/1.73 m^2^) and gradually decrease during adulthood, with a gradual increase in albuminuria (*dashed lines*) into the microalbuminuria (30–300 mg/24 h) or macroalbuminuria (>300 mg/24 h) range. However, renal function may deteriorate much faster and lead to albuminuria in the first decade of life [13] and end-stage renal disease in early adulthood [16]

An obvious reason for a low total nephron number is found in individuals with a solitary functioning kidney (SFK)—for example, due to renal agenesis or aplasia, or in an MCDK. Even though there may be additional nephron formation in the congenital SFK, i.e. when the reduction in renal mass occurs before the termination of nephrogenesis [12], the total number will not reach the full potential and will result in glomerular hyperfiltration. In order to study the effects of glomerular hyperfiltration in humans, we designed the KIMONO study (Kidney of Monofunctional Origin) [13] and were able to show that one-third of children with an SFK at an age of just 10 years already show signs of renal injury, defined as hypertension, albuminuria or the use of renoprotective drugs. Using generalized estimating equations analysis, we found an increase in albuminuria, especially in patients with additional renal tract malformations, including reflux in the SFK [13]. A decrease in GFR can also be seen starting from early teens. In fact, the GFR curve in patients with an SFK resembles the GFR curve in patients with diabetes and subsequent glomerular hyperfiltration [14]. In diabetes, GFR is at first normal or somewhat increased, but as time goes by, microalbuminuria develops, and in a few more years this progresses to macroalbuminuria and the GFR starts to fall (Fig. 2). This ends in chronic kidney disease, and diabetes is in fact the leading cause for renal replacement therapy in adults [15].

And just as diabetic nephropathy ends in renal failure, so may renal function in children with a congenital SFK. A recent long-term follow-up study on patients with several congenital renal tract anomalies showed that 20–40 % of patients with an SFK are in renal failure by the age of 30 years [16]. Unfortunately, there are several methodological issues in these studies that may have had an impact on this poor outcome, such as the small group size, the retrospective collection of data, the presence of additional renal tract anomalies and the likeliness of selection bias as some patients were identified based on the presence of hypertension and/or proteinuria. As this is currently the only study to present data on long-term renal survival in such patient cohorts, it is difficult to estimate true risks for renal failure in individuals with SFK. The least that can be concluded is that a solitary functioning kidney is not always a benign condition without any sequelae and that some patients may end up in renal failure.

Such statements have long been questioned, as most kidney donors are considered to be in good health, even after long-term follow-up [17]. In fact, life expectancy among kidney donors is no different from that of the general population, even though some signs of renal injury do occur in these donors, with an increased incidence of hypertension and proteinuria reported in one long-term study [17]. One might argue that a lower incidence could be expected as kidney donors are screened intensively prior to donation and will therefore represent a relatively healthy segment of the population. The absence of a difference in the incidence of hypertension or end-stage renal disease may, in that view, even be considered another sign of hyperfiltration injury.

However, there are fundamental differences between starting life with one kidney and losing or donating one as an adult. One of the main differences can be found in the degree of glomerular hyperfiltration. Animal studies have shown that nephrectomy during the final stages of kidney development results in a doubling of glomerular hyperfiltration when compared to nephrectomy in early adulthood [18]. As glomerular hyperfiltration is the key to future renal damage [1], this may account for the differences between a congenital and acquired SFK.

Unfortunately, it is currently impossible to predict which individuals with an SFK will suffer from long-erm sequelae. The aim of the KIMONO study is to be able to allow for a differentiation in hyperfiltration injury risk. However, until such predictions have proven to be useful, follow-up of all individuals with a SFK is warranted [19].

## Perinatal programming of nephron endowment

As nephrogenesis takes place entirely before term birth, intrauterine growth restriction is the most widely studied environmental phenomenon influencing renal development [11]. Professor David Barker was one of the first to describe the association between a low birth weight and later cardiovascular deaths [20]. This was followed by an abundance of studies that showed increased risk for diabetes, stroke and hypertension as well [21]. The former Barker hypothesis, now entitled the Developmental Origins of Health and Disease (DOHaD), states that foetal and early postnatal influences or hits program organs and organ systems. This programming effect may have short-term benefits, but results in malfunction in the long run.

In addition to diabetes and cardiovascular events, perinatal programming has been shown to have an effect on the kidney. A lower birth weight, as a marker of restricted intrauterine growth, is associated with a reduced number of nephrons: about 250,000 nephrons fewer are formed when the birth weight is 1 kg lower [22]. Such a decrease in nephron endowment results in an increased risk of renal failure [23]. A recent meta-analysis showed that a low birth weight is associated with a 75 % higher risk of albuminuria, low GFR and chronic kidney disease [24]. Even though the combination of the Barker and Brenner hypotheses offers an explanation for this association, no definitive proof has been found that a low nephron endowment per se causes increased risk for hypertension or renal injury.

Many animal models have been used to study such associations between nephron number, cardiovascular function, renal function and low birth weight [25]. Some of these models, for instance uterine artery ligation, are based on placental dysfunction, which is the main cause of low birth weight in developed countries. Other models have focussed on nutritional programming, for instance through protein restriction of pregnant animals or restriction in total food intake. These latter models have been used the most widely and have shown similar results: a lower nephron number, with higher blood pressure, more albuminuria and glomerulosclerosis and chronic kidney disease in the long run [25]. In fact, a low protein diet fed to pregnant mice drastically reduces the life span of their offspring by an average of 200 days [26].

A human cohort extensively studied for the effects of caloric restriction during pregnancy has been the Dutch Hunger Winter cohort: in the occupied region of the Netherlands, daily rations varied between 400 and 800 calories in the winter of 1944–45, a massive shortage during a specific time frame of pregnancy [27]. All offspring showed altered glucose handling later in life, but the ones exposed to growth restriction during the periods of conception or early gestation specifically showed an increased risk of hypertension and doubled rates of coronary heart disease. The fastest rate of nephrogenesis is during mid-gestation, and those exposed to the famine during that time showed a threefold higher incidence of albuminuria as adults [28]. A recent famine study in Africa has confirmed these results, even at an earlier age [29]. These studies show that caloric restriction is a highly important environmental factor that still affects a large part of the population worldwide. In addition to macronutrient restrictions, deficits in micronutrients have also been shown to negatively influence kidney development, such as vitamin A [30], sodium [31], iron [32], and zinc [33].

Another common condition that affects kidney development is diabetes: dysregulation of glucose is found in one in six pregnancies according to recent criteria of the International Association of Diabetes and Pregnancy Study Groups (IADSPG) [34]. Many consequences of diabetes for renal development have been identified, both in extreme glucose concentrations as well as with just slight alterations. First, nephron endowment is decreased. In an animal study, this reduction was found to be approximately 40 % [35]. Increased apoptosis was described via activation of the intrarenal renin–angiotensin system (RAS) and NF-kappaB and p53 signalling, as well as the generation of reactive oxygen species. Functionally, there is a reduced renal reserve, more albuminuria and higher blood pressure, which can be prevented by abolishing hyperglycaemia during nephrogenesis with insulin [36]. This study illustrates the importance of strict screening for gestational diabetes for future renal health of the offspring.

Maternal diabetes is also associated with CAKUT and with a three-fold increased risk of renal agenesis and dysplasia [37]. It should, however, be noted that familial mutations of HNF1beta were not studied in this study. Mutations in HNF1beta are well known to be associated with renal cysts and diabetes syndrome, which may link maternal diabetes with renal defects in offspring, such as an MCDK [38].

Another environmental factor influencing kidney development is the use of medication by the pregnant mother [39]. Some drugs have been shown to influence renal development, but there is still much research needed to elucidate the effects of the majority of medications on nephrogenesis. One drug that has been studied in more detail is dexamethasone. Normally, a foetus is protected from high levels of maternal steroids through inactivation in the placenta by 11 beta-hydroxysteroid dehydrogenase 2 (11beta-HSD2) [40]. Synthetic steroids circumvent this metabolism, thereby passing through the placenta and promoting the secretion of surfactant in the foetal lung. Unfortunately, negative effects on the kidney are found as well, and many animal studies have shown lower nephron numbers, with hypertension in the long run [41]. The effects of maternal malnutrition and stress may act through this same pathway, as malnutrition has been shown to result in a reduced expression and activity of placental 11beta-HSD2 [42].

Another group of drugs that can have a major impact on renal development are the angiotensin converting enzyme inhibitors and angiotensin receptor blockers, as the RAS is essential for kidney development [43]. Gross disturbance of the RAS may mimic mutations of the RAS genes and result in renal tubular dysgenesis with a reduced number of tubular sections and dedifferentiated tubuli [44]. Another consequence of perinatal angiotensin blockade may just be a lower nephron number, with all its sequelae [45].

## Prematurity

Postnatal drug administration to the prematurely born neonate may have similar effects. Being born before the 36th week of gestation indicates that nephrogenesis has not yet ceased and will continue after birth. Any drug administered to a prematurely born neonate therefore has the potential to disturb normal nephrogenesis, especially before 28 weeks of gestation. Widely used drugs in such infants are antibiotics, such as aminoglycosides, non-steroidal anti-inflammatory drugs for closure of a patent ductus arteriosus and diuretics. All of these drugs have been shown to potentially hamper nephrogenesis (for an overview, see [39]).

In addition to drugs, the majority of premature neonates do not grow well during the first weeks after birth, leading to extra-uterine growth restriction [46]. This has similar effects on the kidney as intrauterine growth restriction as it leads to a reduction in nephron endowment [47]. Whether it is due to the kidney or to other organ systems that are altered after premature birth, a high proportion of hypertension is found: a Dutch follow-up study of premature offspring at the age of 19 years showed that half had a blood pressure in the prehypertensive or hypertensive range [48].

## Perinatal programming of renal tubular function

The consequences of a suboptimal intrauterine environment for tubular function have been studied in less detail than have the glomerular consequences. Most attention has been on sodium transporters, as a possible explanation for hypertension after low birth weight was hypothesized to be caused by altered renal sodium handling. In fact, an upregulation of NKCC2 was found after protein restriction, leading to increased renal sodium retention [49]. Other tubular transport systems have been shown to be altered as well, including a defect in tubular phosphate transport [50]. Maternal low protein diet in rats, as a model for intrauterine growth restriction, has been shown to lead to increased urinary calcium excretion [51], whereas magnesium transport was not affected [52].

## Conclusion

Maternal malnutrition, stress or diseases such as diabetes, uteroplacental insufficiency, maternal and neonatal drugs and premature birth with growth restriction are environmental factors with the potential to hamper kidney development. This may result in gross anomalies, such as renal agenesis or multicystic dysplasia, but most effects are more subtle and lead to a reduction in nephron endowment. Starting life with a reduced nephron endowment starts a vicious cycle that leads to hyperfiltration, hypertension and/or glomerular damage with albuminuria, and may end in chronic kidney disease. Acknowledging these health risks and following up on patients longitudinally is essential to reduce the associated risks. In addition, more research is needed to evaluate factors that may hamper nephrogenesis and study the pathways that are involved in this programming, all aimed at preventing exposure to such factors or limiting the negative effects by studying rescue therapies. Until science has developed that far, patients should be offered structured follow-up to find and treat risk factors for future health.

## Key research points

- Development of in vivo imaging of glomeruli may allow for the individual determination of nephron numbers, and thereby the study of the consequences of a low nephron endowment
- Studies into the effects of medications administered during nephrogenesis, either in pregnant women or in prematurely born neonates, will allow for the prevention of low nephron endowment and associated long-term sequelae
- Further unravelling of the molecular regulation of kidney development may allow for the identification of key (growth) factors that can be used as a rescue therapy when kidney development is hampered.

## Summary points

- A low nephron endowment leads to glomerular hyperfiltration, associated with hypertension, glomerular damage and proteinuria, and starts a vicious cycle that ends in renal failure in the long run.
- Nephron endowment is influenced by genetic and environmental factors, such as maternal malnutrition, stress or diseases, such as diabetes, uteroplacental insufficiency, maternal and neonatal drugs and premature birth.
- A solitary functioning kidney is an example of low nephron endowment and is associated with hyperfiltration injury in up to one-third of patients, and may result in end-stage renal disease.

## Questions (answers are provided following the referrences)

1. Which factor is the best marker to predict nephron number in a 22-year-old adult?
  - Renal size on ultrasound
  - Birth weight and gestational age
  - Blood pressure level
  - Glomerular filtration rate
  - Level of proteinuria
2. Which statement(s) are true concerning nephron numbers?
  - On average, a little under 1 million nephrons per kidney are formed, with a small inter-individual range.
  - Nephrons are only formed during gestation.
  - Both genetic as well as many environmental factors are important in determining final nephron endowment.
  - All three answers are correct.
3. A patient with a congenital solitary functioning kidney…
  - Has a condition that warrants a relatively infrequent but long-term follow-up.
  - Can be safely discharged from follow-up when check-ups at the age of 2 years are normal.
  - Should be checked until the age of 10 years, as no long-term sequelae have been noted after that.
  - Has sufficient renal reserve capacity to go through life without any expected renal consequences, just as has been shown for large numbers of kidney donors.
4. Drug use during kidney development…
  - is safe, unless noted differently in the package leaflet.
  - may have a wide variety of consequences.
  - should always be prevented.
  - should be subject of more research.
  - (b) + (d)
5. The effect of gestational diabetes on nephrogenesis…
  - is characterized by renal hypoplasia and/or dysplasia.
  - may be found within the “normal” range of glucose levels.
  - may be explained by a genetic mutation in HNF1beta, which is associated with Maturity Onset Diabetes of the Young (MODY) and renal hypodysplasia.
  - all of the above.
